# Quantifying changes over 1 year in motor and cognitive skill after transient ischemic attack (TIA) using robotics

**DOI:** 10.1038/s41598-021-96177-y

**Published:** 2021-08-23

**Authors:** Leif E. R. Simmatis, Stephen H. Scott, Albert Y. Jin

**Affiliations:** 1 Toronto Rehabilitation Institute, University Health Network, Toronto, ON Canada; 2 Centre for Neuroscience Studies, Queen’s University, Kingston, ON Canada; 3 Department of Biomedical and Molecular Sciences, Queen’s University, Kingston, ON Canada; 4 Department of Medicine (Neurology), Queen’s University, Kingston, ON Canada

**Keywords:** Cerebrovascular disorders, Migraine, Human behaviour, Diseases of the nervous system

## Abstract

Recent work has highlighted that people who have had TIA may have abnormal motor and cognitive function. We aimed to quantify deficits in a cohort of individuals who had TIA and measured changes in their abilities to perform behavioural tasks over 1 year of follow-up using the Kinarm Exoskeleton robot. We additionally considered performance and change over time in an active control cohort of migraineurs. Individuals who had TIA or migraine completed 8 behavioural tasks that assessed cognition as well as motor and sensory functionality in the arm. Participants in the TIA cohort were assessed at 2, 6, 12, and 52 weeks after symptom resolution. Migraineurs were assessed at 2 and 52 weeks after symptom resolution. We measured overall performance on each task using an aggregate metric called Task Score and quantified any significant change in performance including the potential influence of learning. We recruited 48 individuals to the TIA cohort and 28 individuals to the migraine cohort. Individuals in both groups displayed impairments on robotic tasks within 2 weeks of symptom cessation and also at approximately 1 year after symptom cessation, most commonly in tests of cognitive-motor integration. Up to 51.3% of people in the TIA cohort demonstrated an impairment on a given task within 2-weeks of symptom resolution, and up to 27.3% had an impairment after 1 year. In the migraine group, these numbers were 37.5% and 31.6%, respectively. We identified that up to 18% of participants in the TIA group, and up to 10% in the migraine group, displayed impairments that persisted for up to 1 year after symptom resolution. Finally, we determined that a subset of both cohorts (25–30%) experienced statistically significant deteriorations in performance after 1 year. People who have experienced transient neurological symptoms, such as those that arise from TIA or migraine, may continue to experience lasting neurological impairments. Most individuals had relatively stable task performance over time, with some impairments persisting for up to 1 year. However, some individuals demonstrated substantial changes in performance, which highlights the heterogeneity of these neurological disorders. These findings demonstrate the need to consider factors that contribute to lasting neurological impairment, approaches that could be developed to alleviate the lasting effects of TIA or migraine, and the need to consider individual neurological status, even following transient neurological symptoms.

## Introduction

Transient ischaemic attack (TIA) is defined as transient neurological symptoms of suspected vascular origin that resolve on their own without leaving clear evidence of brain tissue injury^1^. The self-recovery aspect, for decades, made researchers and clinicians alike think that TIA was associated with no lasting brain dysfunction for the individual compared to stroke, which is pathophysiologically related. We now appreciate that TIA, as with stroke, can lead to cognitive and motor deficits in some individuals^2–6^. However, what is not clear is whether motor and cognitive impairment in this clinical population change over time.

Traditional clinical measures have been used to quantify changes primarily in cognition after TIA or minor stroke, but less attention is paid to motor impairments or recovery. It is well-known that people who have had TIA or minor strokes experience cognitive impairments, mood decline, and reduced quality of life after symptom resolution^4, 5, 7–9^. Some individuals will improve between 7 days and 1-month post-event^10^; however, by 3- to 6-months, some individuals may still have impairments in cognitive domains such as information processing^11^. Impairments in verbal memory could persist for as much as 3 years post-event in some people^12^. However, the same detailed characterization does not exist for possible changes to motor-related ability after TIA. Potentially, an approach that considers cognitive and motor aspects of behaviour could shed light on how performance in these behavioural domains changes over time in people who have had TIA.

We have previously demonstrated that people who have had TIA can have deficits in cognitive and motor tasks in an objective robotic assessment paradigm for up to 2 weeks after symptom resolution^2^. However, it is presently unclear whether or not these individuals will have persistence of *both* cognitive and motor deficits for months after their events. At this point, persistence of cognitive deficits has been suggested to occur in a subset of individuals on traditional cognitive assessments^3, 10, 13^. Our objective in this study was to quantify changes in both motor and cognitive functionality over time in a cohort of individuals who had TIA and were seen initially within 2 weeks of symptom resolution. We previously reported on part of this cohort^2^, and in this work we report on an expansion of it that utilizes the Kinarm robotic assessment platform and its associated Kinarm Standard Tests™ (KSTs) to perform behavioural assessments. Furthermore, we investigated an active control group of individuals who experienced transient neurological symptoms but were diagnosed with migraine. These individuals were included because migraine is frequently confused with TIA^14^, even though they are pathophysiologically distinct. Thus, migraineurs make a good comparison group to assess whether any changes over time observed in the TIA group were specific to that group.

## Results

### Participants and demographics

We recruited 48 people who had TIA and 28 people who had migraine. The median [IQR] age of the participants at the first assessment was 69.4 [15.3] years in the TIA cohort and 60.0 [14.5] years in the migraine cohort. See Table 1 for a brief demographic summary of the participants in the study. We quantified the NIHSS in people who had TIA and people who had migraine. In the TIA cohort, two individuals had NIHSS scores > 0. One individual had a score of 2, because of left leg drift and left facial droop, however their diffusion-weighted magnetic resonance imaging (DWI) scan was negative for a corresponding lesion. Another individual had an NIHSS of 4 because of diabetic lumbosacral radiculoplexus neuropathy, which pre-existed their TIA. In both instances, the cause of the increased NIHSS score was not related to the TIA in question (no MRI evidence of corresponding lesions) and therefore we allowed these participants to be included in the final analysis. In the migraine cohort, one individual had an NIHSS = 1. In this case, the individual had right side face and arm numbness and dizziness; their CT and MRI scans were both normal.

**Table 1:** Participant demographics.

	TIA	Migraine
N	48	28
Age median [IQR]^a^	69.4 [15.3]	60.0 [14.5]
% Right-handed^a^	93	94
% Female^a^	46	54
NIHSS (n > 0, [scores])	2, [2, 4]^†^	1, [1]^b^

^a^Z-scores used in classification analyses are adjusted for age, sex, and handedness, and so statistical differences in age or proportion of sex or handedness were not tested for.

^b^NIHSS scores > 0 were permitted in these instances because the scores were not related to the event in question.

### Group-level statistical tests of Z-Task Scores in TIA and migraine

**Figure 1:**
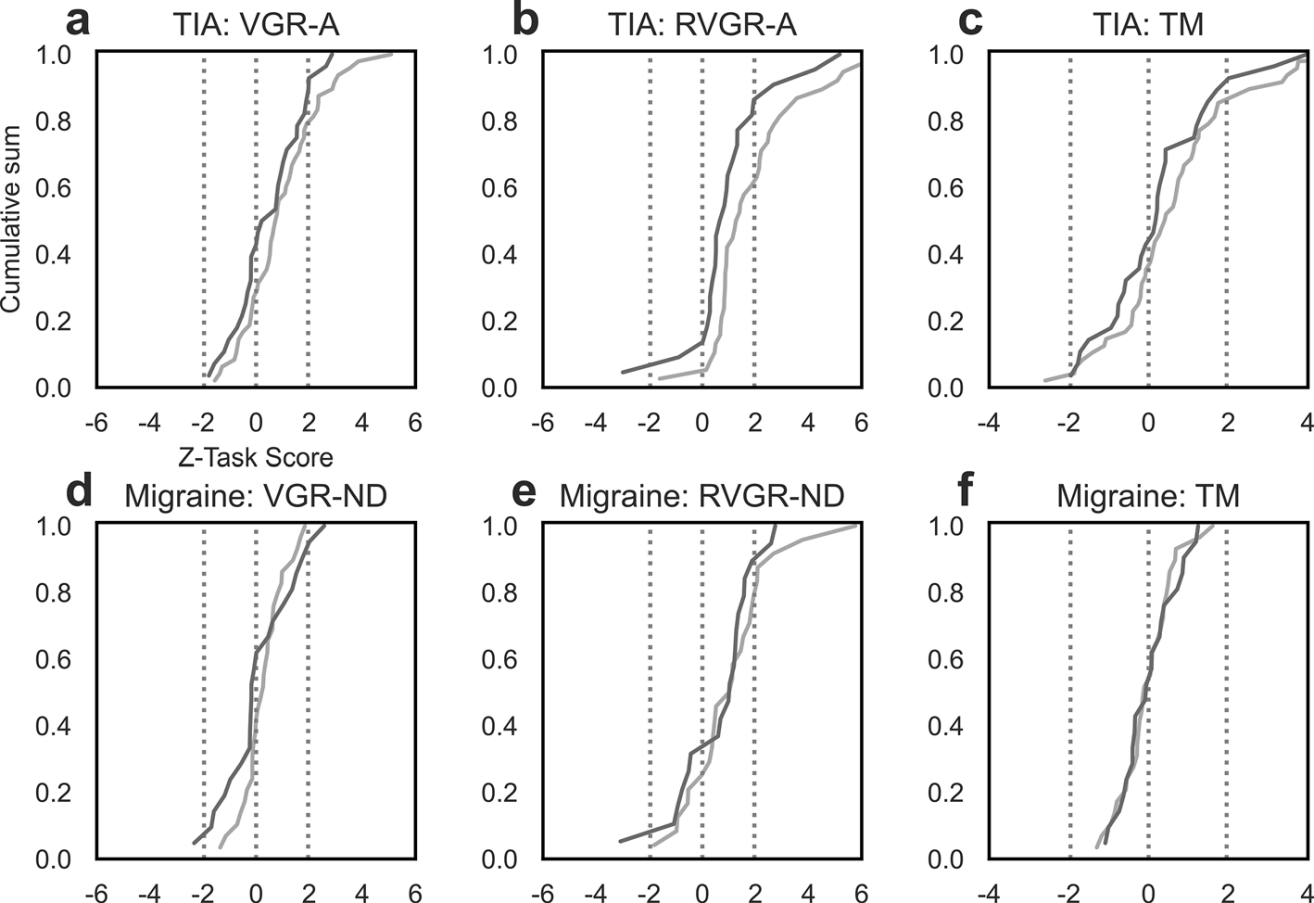
Cumulative sums of Z-Task Scores in TIA and migraine cohorts at first and last assessments. (**a–c**) Z-Task Score distributions for the TIA cohort in VGR-A, RVGR-A, and TM tasks. Dashed vertical lines indicate ± 1.96 (peripheral lines) or 0 (central line). Assessments within the first 2 weeks of symptom resolution are plotted in light grey, and those at 1 year after symptom resolution are plotted in dark grey. (**d–f**) The same as (**a–c**), but for migraineurs. Tasks were VGR-ND, RVGR-ND, and TM. Assessments within 2 weeks of symptom resolution are plotted in light grey, and assessments from 1 year after symptom resolution are plotted in dark grey.

We tested the hypotheses that TIA- and migraine cohorts’ Task Scores were significantly different from 0, and the hypothesis that the difference between first- and last assessments was significantly different from 0, using 1-sample Student’s t-tests. See Table 2 for a summary of p-values and test statistics for these tests. Furthermore, Fig. 1 displays the cumulative sums of Z-Task Scores for three tasks at first and last assessments for each group; these provide a depiction of the location and scale of these distributions to provide further context to our statistical tests.

**Table 2:** Student’s t-test results comparing Z-Task Score distributions in TIA and migraine.

	TIA						Migraine					
	2 weeks		1 year		Difference		2 weeks		1 year		Difference	
	p	t	p	t	p	t	p	t	p	t	p	t
VGR-D/UA	2.7e−5^†^	4.65	0.18	1.37	0.01	2.70	0.01	2.74	0.91	0.11	0.07	1.94
VGR-ND/A	2.9e−5^†^	4.63	0.05	2.08	0.08	1.81	0.07	1.88	0.76	0.31	0.63	0.48
BOB	5.4e−6^†^	5.31	0.02	2.49	0.03	2.30	0.05	2.05	0.23	1.25	0.24	1.22
OH	0.43	0.79	0.35	0.95	0.12	− 1.61	0.93	0.09	0.08	1.86	0.38	− 0.89
OHA	0.08	1.81	0.03	2.36	0.15	− 1.50	0.55	0.60	0.44	0.79	0.59	− 0.54
RVGR-D/UA	9.7e−7^†^	5.83	0.01	2.83	4.3e-4^†^	4.17	2.0e-4^†^	4.30	0.02	2.59	0.01	2.83
RVGR-ND/A	4.8e−7^†^	6.09	0.01	2.84	7.9e-3	2.94	4.3e-3^†^	3.17	0.07	1.91	0.15	1.49
TM	0.01	2.65	0.35	0.95	0.88	0.15	0.80	− 0.25	0.91	0.12	0.63	− 0.48
SPS	3.9e−3^†^	3.08	0.54	0.63	0.39	0.89	0.42	0.83	0.87	0.13	0.77	0.30
APM-ND/A	0.04	2.08	0.98	0.03	0.02	2.41	0.02	− 2.51	0.17	− 1.48	0.84	0.21

*ND* non-dominant arm (migraine only), *D* dominant arm (migraine only), *UA* unaffected arm (TIA only), *A* affected arm (TIA only), *t* t-statistic.

Small values are represented using scientific notation for compact formatting.

^†^p < (0.05/60).

At the group level, tasks performed by the TIA cohort captured frequent differences from 0 (standard normal mean) at 2-weeks, although such differences were uncommon at 1-year, and the change over time often did not differ significantly from 0. See Fig. 1 and Table 2 or graphical and numerical summaries of these results. Consider the example of RVGR-A: a significant difference from 0 was observed at 2-weeks (p = 4.8e−7; significant after multiple comparison correction), but there was no significant difference from 0 by 1-year, and the group-level difference between 2-weeks and 1-year was not significantly different from 0. Similar observations were made in other tasks, with the only exception being RVGR-UA. In this task, a significant difference from 0 was observed at 2-weeks but not at 1-year; the difference between assessments was significantly different from 0.

In the migraine group, fewer significant differences and changes were observed than in the TIA group. Only RVGR-D had a group mean that was significantly different from 0 at 2-weeks, and no tasks had significant differences from 0 at 1-year. Furthermore, none of the differences between assessments was significant.

### Impairment rates in TIA and migraine

We collected KST data at 2 weeks and 1 year after symptom resolution in both TIA and migraine groups, as well as at 6-weeks and 3-months in the TIA group. We identified that tasks that were used for testing the integrated cognitive-motor ability identified the greatest number of impairments in both cohorts and that, at a group level, the overall impairment rate appeared to decline in most tasks. See Table 3 for a summary of these results. Individuals in the TIA cohort demonstrated frequent impairments on multiple behavioural tasks spanning motor and cognitive domains. RVGR-UA identified 51.3% of people in the TIA cohort as impaired within 2-weeks, which reduced to 27.3% at 1 year.

**Table 3:** Impairment rates in TIA and migraine cohorts at all assessment time points.

Impaired/total (percent)	TIA				Migraine	
	2 weeks	6 weeks	3 months	1 year	2 weeks	1 year
VGR-D/UA	15/48 (31.2)	8/44 (18.2)	7/39 (17.9)	4/28 (14.3)	3/29 (10.3)	1/21 (4.8)
VGR-ND/A	14/48 (29.2)	9/43 (20.9)	9/39 (23.1)	6/28 (21.4)	2/29 (6.9)	3/21 (14.3)
BOB	8/38 (21.1)	4/34 (11.8)	2/30 (6.7)	1/20 (5.0)	3/23 (13.0)	3/16 (18.8)
OH	1/48 (2.1)	2/44 (4.5)	3/39 (7.7)	2/28 (7.1)	2/29 (6.9)	3/21 (14.3)
OHA	4/48 (8.3)	3/44 (6.8)	4/39 (10.3)	4/28 (14.3)	0/29 (0.0)	2/21 (9.5)
RVGR-D/UA	21/39 (51.3)	13/36 (36.1)	8/31 (25.8)	6/22 (27.3)	9/24 (37.5)	6/19 (31.6)
RVGR-ND/A	16/38 (42.1)	10/35 (28.6)	7/31 (22.6)	5/22 (22.7)	8/24 (33.3)	3/19 (15.8)
TM	10/48 (20.8)	3/44 (6.8)	6/39 (15.4)	4/28 (14.3)	0/29 (0.0)	0/21 (0.0)
SPS	7/38 (18.4)	2/34 (5.9)	2/29 (6.9)	0/19 (0.0)	0/23 (0.0)	0/18 (0.0)
APM-ND/A	6/47 (12.8)	2/43 (4.7)	2/36 (5.6)	1/27 (3.7)	0/18 (0.0)	0/12 (0.0)

*ND* non-dominant arm, *D* dominant arm, *UA* unaffected arm, *A* affected arm.

Tasks testing cognition and motor behaviour separately also captured frequent impairments. In TM (a cognitive task), over 20% of individuals in the TIA cohort were impaired at the first assessment, which was approximately 15% at the end of 1 year of follow-up. In VGR (a motor task), over 30% of the group was impaired within 2-weeks on this purely sensorimotor task, which reduced to ~ 14% by the end of 1 year of follow-up.

Individuals in the migraine group were most-often impaired on tasks testing cognitive-motor integration. For example, 37.5% of individuals in the migraine cohort had impairments in RVGR-D. This reduced to 31.6% at 1 year. At the first assessment, two other tasks that tested motor skill specifically had impairment rates > 10%: BOB (3/23; 13.0%) and VGR-D (3/29; 10.3%). At the second assessment, the impairment rate on BOB was 3/16 (18.0%) whereas the impairment rate on VGR-D was 1/21 (4.8%).

### Individual longitudinal changes

**Figure 2:**
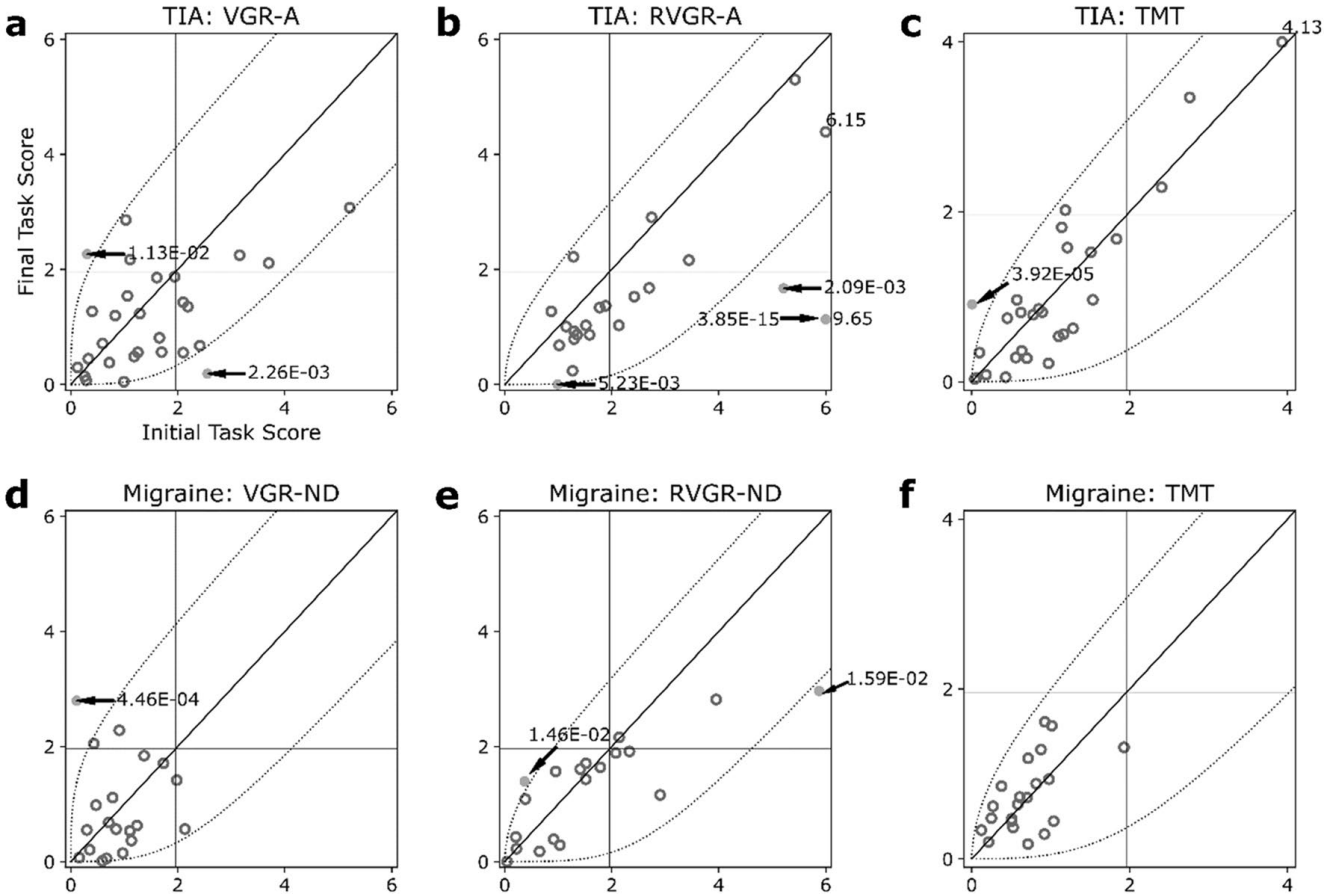
Scatterplots of one-sided Task Scores at first- and second assessments in TIA (**a–c**) and migraine (**d–f**) cohorts. All: Horizontal and vertical dashed lines indicate impairment thresholds (1.96); diagonal lines indicate unity. Curved dashed lines indicate thresholds of significant change based on the expected change of healthy control participants, accounting for learning effects in RVGR-A/RVGR-ND and TM only. Values > 6 (VGR-A/VGR-ND, RVGR-A/RVGR-ND) or > 4 (TM) are marked on the plots (no arrows pointing at relevant datapoints). Individual significant change p-values are marked when changes were significant (arrows pointing at relevant datapoints). Open darker circles represent non-significant changes (i.e., within the curved boundaries), whereas filled circles represent significant changes (i.e., outside the curved boundaries). (**a–c**) Plots for TIA. (**d–f**) Plots for migraine.

Next, we considered changes in score between first and last assessments at an individual level. See Fig. 2 for scatterplots of first- and last-Task Score plots, with p-values for individual changes labelled when changes between assessments were statistically significant.

Figure 2a–c depicts scatterplots for the TIA group. At an individual level, most individuals did not change significantly between assessments, reflecting the results of the group-level analyses above. However, there were a few individuals who changed significantly, demonstrating the heterogeneity of this cohort, and which are not reflective of the group-level results. Furthermore, changes were bidirectional; deterioration and improvement both occurred. In VGR-A (Fig. 2a) seven individuals were impaired at 2-weeks and of those participants, three remained impaired at 1-year (in the upper-right quadrant of the scatterplot). Therefore, 10.7% of individuals remained impaired in this task at both timepoints. Only two individuals demonstrated statistically significant changes across assessments in VGR-A: one individual worsened and the other improved. In the TM task (Fig. 2c), 3 individuals had impairments at 2-weeks and those individuals stayed impaired at 1-year (10.7%). Furthermore, 1 individual deteriorated and 0 improved in TM. Finally, in RVGR-A, 4/22 participants (18.2%) had impairments at both timepoints and 3 improved significantly (none deteriorated significantly). Note that in all three example tasks mentioned here, group-level difference between assessments was not significantly different from 0, see Table 2.

In the migraine group (Fig. 2d–f), few individuals demonstrated persistence of impairments and, as a whole, the group generally stayed within the limits of significant change. Furthermore, as in the TIA cohort, we observed a small number of individuals who changed significantly between assessments, even if the corresponding group effects were not significantly different from 0. For example, there were no instances of someone in the migraine group being impaired at both first- and last assessment in either VGR-ND or TM. There were three instances of this in RVGR-ND (3/21, i.e., 14.3%), one of which had impairments very close to the threshold (Task Scores of ~ 2.0). Very few individuals in this group showed any significant change; 1 in VGR-ND, 2 in RVGR-ND, and 0 in TM.

### Patterns of significant change across tasks in TIA and migraine

**Figure 3:**
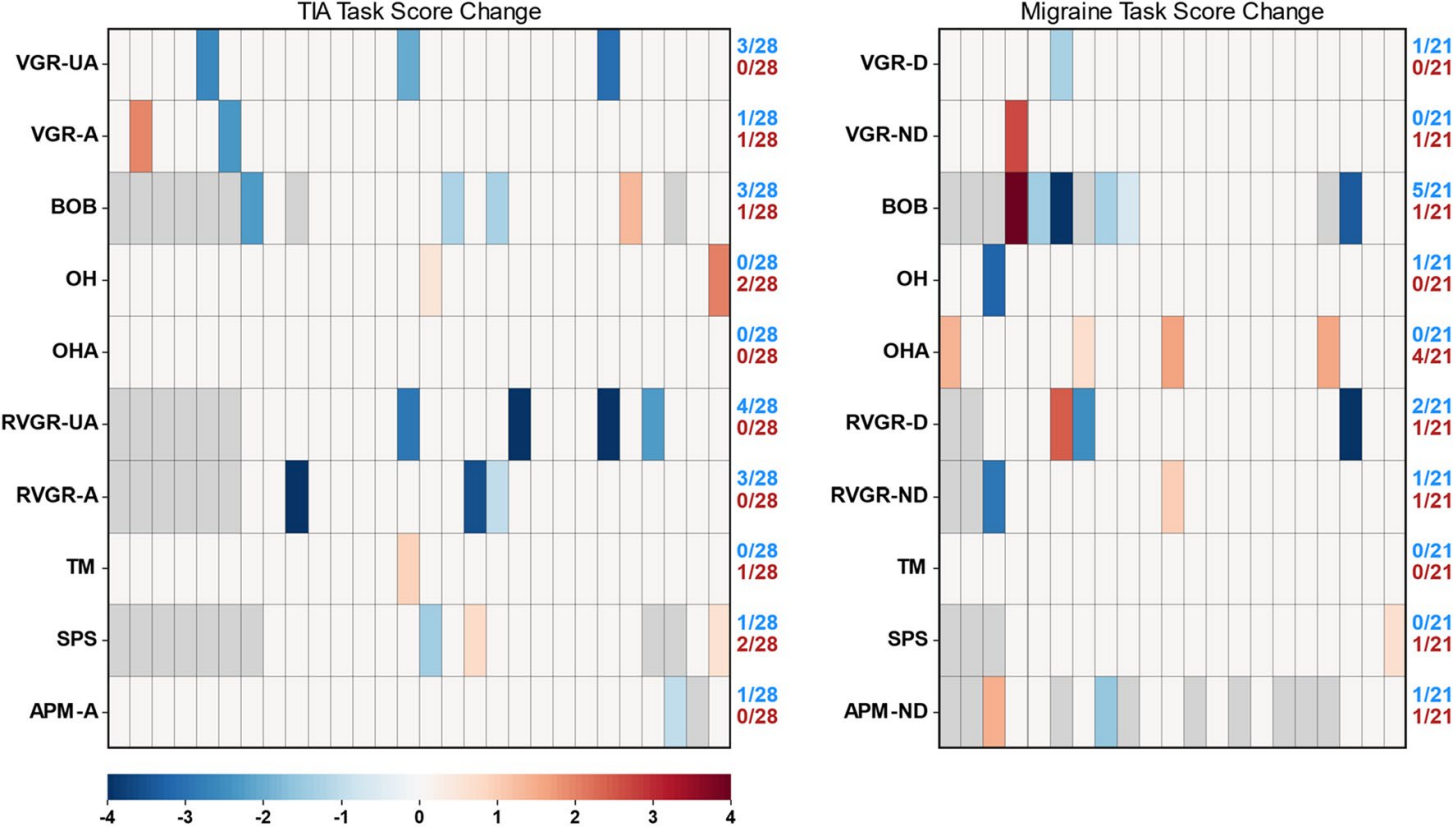
Heatmaps of significant changes in TIA and migraine groups between 2-weeks and 1-year. Participants are arrayed along the x-axes in no particular order. -*A* affected arm, -*UA* unaffected arm, -*D* dominant arm, -*ND* non-dominant arm. Blue cells represent significant lowering (improvement) in Task Score between 2 weeks and 1 year. Red cells represent significant increase (deterioration) in Task Score between 2 weeks and 1 year. Gray cells indicate missing values for those participants. Fractions improved and deteriorated are represented on the right-hand side of each axis for each task.

We next investigated the number of significant changes over time across tasks at an individual level. These results are summarized in Fig. 3. For the TIA group, a total of 10 assessments were considered for 28 individuals who completed first and last assessments, i.e., 28 × 10 = 280 assessments were performed. Of these, 23 significant changes were observed, a rate of approximately 1/12 assessments. Furthermore, 16/28 (57%) individuals changed significantly on at least 1 behavioural task, and 6/28 (21%) changed significantly on at least 2 behavioural tasks. Of these, 6/28 (21%) significantly deteriorated on at least 1 task, whereas 13/28 (46%) significantly improved on 1 task. These changes were heterogeneous. For example, one individual in the TIA cohort significantly improved on both BOB and RVGR-A. Another significantly improved in VGR-UA and RVGR-UA, but significantly deteriorated on TM.

In the migraine group, 21/210 total assessments demonstrated significant change between first and last test (approximately 1/10 individuals). Furthermore, 12/21 (57%) changed significantly on at least 1 behavioural task, and 7/21 (33%) changed significantly on at least 2 behavioural tasks. Of these, 8/21 (38%) significantly deteriorated on at least 1 behavioural task, and 7/21 (33%) significantly improved on at least 1 task. As in the TIA cohort, these changes were heterogeneous. For example, one individual got better on RVGR-D, but deteriorated on OHA. Another significantly deteriorated on BOB and VGR-ND.

### Individual change over multiple assessments in TIA

**Figure 4:**
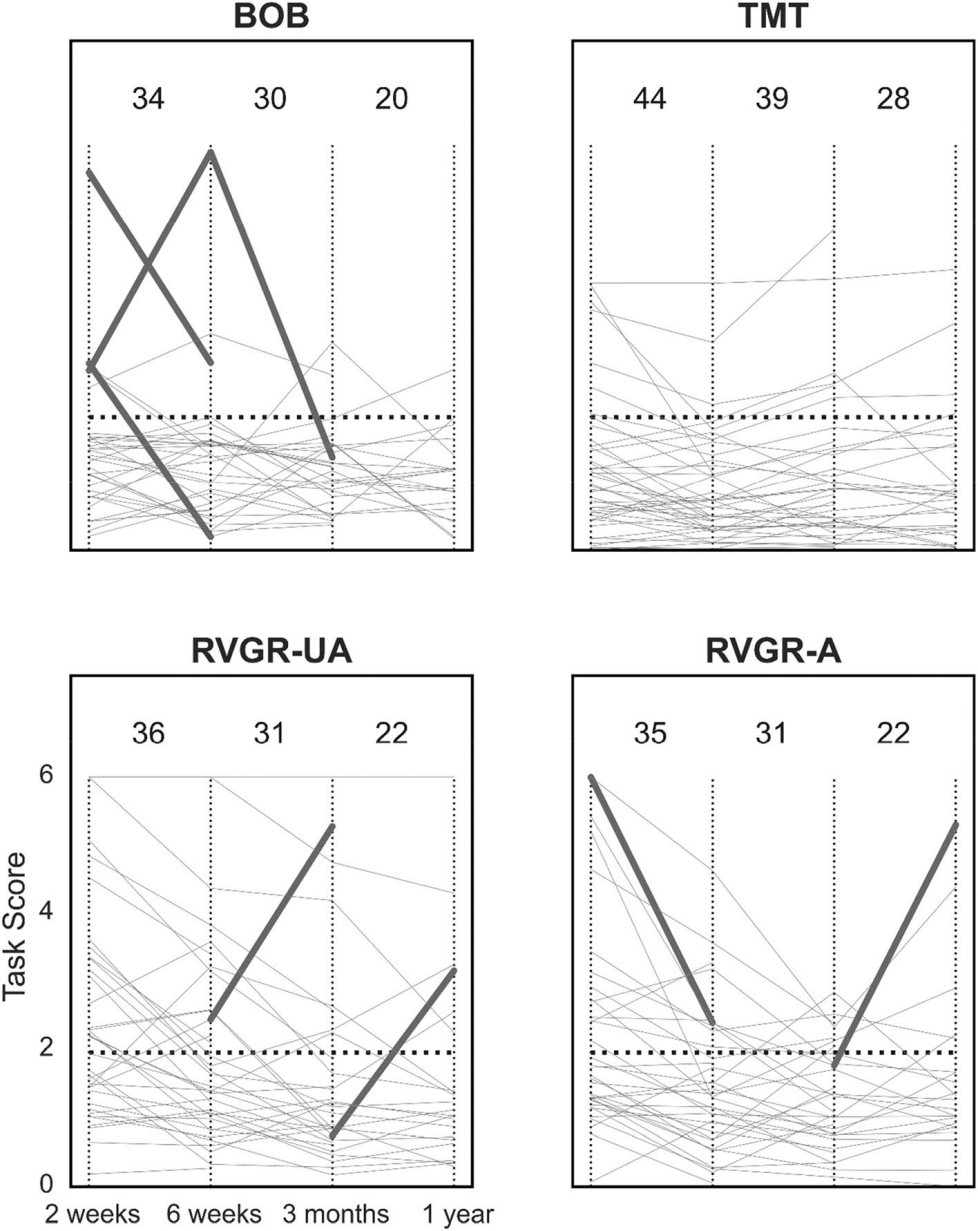
Line plots of task scores over time in the TIA cohort at all 4 assessments with significant changes corrected for the false discovery rate (FDR). *A* affected arm, *UA* unaffected arm. Thin vertical lines demarcate different assessment points for ease of viewing. Horizontal dashed lines represent the threshold for impairment in Task Scores (i.e., > 1.96). Thin grey lines represent each individual’s performance over time. Thick lines indicate significant improvement or deterioration between assessments. Values at the top of each plot indicate the total number of individuals plotted on the relevant interval, e.g., BOB between the first two vertical dashed lines has 34 individuals plotted, one of whom significantly improved, and one of whom significantly deteriorated.

We determined the frequency of Task Score changes between all assessments for the TIA group, accounting for the large number of statistical tests. A subset of these results is depicted in Fig. 4. We generally found that individuals in the TIA group did not change significantly between individual assessments, and sometimes an individual remained impaired throughout follow up (grey lines in Fig. 4 that were higher than the 1.96 Task Score impairment threshold). For example, 2 individuals in RVGR-UA had consistently impaired scores throughout follow-up (see grey lines towards the top of the RVGR-UA panel in Fig. 4).

After accounting for the false discovery rate (FDR), only a relatively small number of Task Score changes between time points in the TIA group were considered statistically significant. Some significant changes had a very high magnitude, such as one individual in BOB that changed > 4 Task Score units between 6 weeks and 3 months. Other significant changes were smaller, but still considered significant when starting values were closer to 0. This reflects the nonlinearity of the Task Score change as a function of the magnitude of the Task Score itself; the further from 0 a value is, the more it has to change in order to be considered significant. This effect is evident from the curvature of the significant change thresholds depicted in Fig. 2.

## Discussion

We performed a longitudinal robotic assessment of people who had TIAs as well as people who had migraines, and found that up to 18% of individuals in the TIA cohort had impairments on behavioural tasks that lasted for 1 year. A persistent impairment rate of up to 10% was observed in the migraine (active control) group. Furthermore, 25–30% of individuals from each group had significantly poorer performance after 1 year on at least 1 task. We observed few group-level significant changes over time (i.e., changes in score that were significantly different from 0), as a consequence of the heterogeneity of individual-level changes. Finally, we observed diverse patterns of change across multiple tasks, which demonstrates the heterogeneous behavioural presentations of TIA and migraine.

Cognitive deficits are known to be associated with TIA and migraine^3, 15–19^, but persistence of impairments in motor tasks and changes in the ability to perform them are less well-characterized. Here, we identified that primarily the TIA group displayed impairments in motor-domain tasks that lasted for 1 year. For example, VGR, a task that straightforwardly tests the ability to make reaching movements with veridical visual feedback, captured more impairments in the TIA cohort than might be expected based on the clinical definition of a TIA. In their affected arms, 3/28 individuals in the TIA group had impairments on VGR that persisted for 1 year. Furthermore, 3/28 individuals transitioned from being unimpaired at 2 weeks to impaired at 1 year. The migraineurs appeared to have less prevalent impairments across all tasks, including from the motor domain, and they also generally did not demonstrate persistence of impairments over the 1 year of follow-up (RVGR-ND was a notable exception to this rule, see Fig. 2e).

We observed persistence of impairment on cognitive- and cognitive-motor tasks in both the TIA and migraine groups that would not be expected based on the clinical definitions of these conditions and the performance of healthy individuals. It is now acknowledged that both stroke and TIA leads to persistent cognitive decline for several years afterwards^10, 20–22^, which can have heterogeneous trajectories^23^ and which are potentially related to post-ischemic immune response^24^ and existing vascular risk factors^25^. Previous work identified that people who have TIAs experience increased rates of whole-brain atrophy beyond that expected for healthy aging^26, 27^. In the present study, 18% of the TIA group had impairments that lasted for more than 1 year on RVGR-A (see Fig. 2b). RVGR previously demonstrated strong learning effects in healthy individuals^28^, and so it is clear that not only do some people in the TIA cohort display deficits in this task, but some fail to learn or improve in the task even after 4 repeated trials. RVGR requires participants to override or inhibit prepotent motor plans to reach towards a visual target, and instead move in the opposite direction to attain a spatial goal, and retain this reversal rule across trials. Healthy adults have been shown to retain motor skills for periods ranging from 1 week^29^ to 1 year^30–32^. Neurological disease, such as stroke, can lead to impaired learning of motor skills such as visuomotor rotations^33^ as well as specific movement features (e.g., initial movement and final accuracy), in a manner dependent upon lesion location^33, 34^. Potentially, white matter injury can influence the rate of visuomotor adaptation in healthy individuals^35^, and can impair executive functioning^36, 37^. In addition to findings in the TIA group, we observed several cases of impairments and persistence of deficits in the migraineurs, with potential causes including disruptions of thalamic sensory relays and impaired thalamocortical connectivity^38, 39^ that could disrupt cortical information processing loops.

Deficits in almost all tasks were less prevalent in the migraine group than the TIA group, which may reflect patterns of post-TIA effects on the brain. For example, in the migraine group at the first assessment, 4/10 tasks did not identify anyone as impaired whereas in the TIA group every task highlighted at least a few impairments. After 1 year, 3/10 tasks failed to capture any impairments in the migraineurs, and these tasks had also not identified deficits at 2 weeks. In contrast, individuals from the TIA group had some impairments at almost every task at both time points (except for SPS at the 1-year assessment). These results are even more remarkable when considering that the TIA cohort was assessed 4 times over the 1 year of follow up, whereas the migraineurs were only assessed twice. Thus, people in the TIA cohort demonstrated poorer group-level performance despite having the potential to benefit from additional practice, even in tasks such as RVGR and TM that have learning effects^28^. The breadth of motor and cognitive-motor impairments in the TIA group could reflect distributed brain pathology. Possible culprits of motor impairments in the TIA group are elevated white matter lesion load^40, 41^, impaired connectivity between motor regions^42^, and damage to the corticospinal tract (or, more broadly, internal capsule)^43–47^, which can affect unimanual and bimanual motor skill. White matter injury can also impair attention^48^, which could impact performance on cognitive-motor tasks such as RVGR.

Some of the most important general observations from this study were that it was not sufficient to consider only group effects when dealing with conditions as heterogeneous as TIA and migraine, and that longitudinal assessment lent additional strength to our investigation of impairments in these groups. We observed very few significant group-level changes over time, i.e., group-averaged changes of score that were significantly different from 0, in both TIA and migraine groups. Yet, we observed that unexpectedly large proportions of individuals from both groups (more so with the TIA group) had significant changes at an individual level. Fifty-seven percent of individuals in both groups significantly changed on at least one behavioural task, and approximately one-third did so on at least two tasks. It is clear from inspecting the data that the disparity between group-level and individual results arose from the bidirectionality of changes. There was little in the way of an average effect across the groups, because the average effect was obfuscated by the presence of both improvements and deteriorations at an individual level. Furthermore, we observed that considering only group-level impairment rates did not convey the true persistence of impairments within individuals. This reflects the value added by considering longitudinal change within individuals instead of only changes in cross-sectional statistics^49, 50^. For example, we observed that 21/39 (51.3%) of individuals in the TIA group were impaired in RVGR-UA at the first assessment, and 6/22 (27.3%) were impaired in this task at the final assessment. However, of the 6 individuals who were impaired at 1-year, 4 had been impaired since the beginning of the study. A similar observation was made in the TMT task; the group impairment rate from 2-weeks to 1-year decreased from 20.8 to 14.3% (a 50% relative decrease), but of the 4 individuals that had impairments at the 1-year assessment, 3 had been impaired at the 2-week assessment, demonstrating persistence of impairment at an individual level. It is important to address this dichotomy between the apparent group-level decrease in impairment and the persistence of impairments at an individual level. In the present study, considering only impairment rates at a single time point, this valuable information is lost: these individuals had long-term impairments in cognitive-motor integration for up to 1 year after TIA. Future studies with larger samples, particularly at longitudinal follow-ups, will be important to grasp the extent of cognitive and/or motor impairment persistence after TIA, and any factors that contribute to it.

### Limitations

Our study has some limitations to address. First, although we discuss significant change, our previous work did not consider inter-test intervals longer than approximately 2 weeks. Thus, it remains unclear how much change is permissible over longer time scales and if, indeed, those results generalize. We did observe subjective differences between TIA and migraine cohorts after 1 year of follow-up on some tasks such as VGR and TM, although the sample sizes at 1 year were quite small. The sample size reduced over 1 year largely because participants lost interest, or moved away and so were unable to participate any further. Further work with larger sample sizes will be required to directly compare these two clinical populations, particularly using longitudinal data. It remains possible that because the people in the TIA cohort were seen more frequently than the migraineurs, that they could have had extra practice on tasks that predisposed them to improve more than the migraineurs, especially on tasks with strong learning effects like RVGR. However, we did not identify that individuals in the TIA group improved frequently in the other tasks that also displayed strong learning effects (TM and SPS). Indeed, approximately 10–15% of individuals displayed persistence of impairments *despite* having 4 repeated tests. Furthermore, it is unclear how consistent learning effects are across multiple repeated assessments. Our previous work did not quantify learning effects beyond two assessments. This will require further study, possibly considering RVGR performance changes within-assessment.

### Conclusions

We performed a novel longitudinal assessment of people who had TIA, considering changes over a total of 4 assessments. We also assessed an active control group of migraineurs at 2-weeks and 1-year. We identified first that our TIA cohort had prevalent impairments in motor and cognitive tasks, some of which persisted for up to a year after symptom resolution. We identified substantial heterogeneity across individuals in both cohorts, in terms of impairments as well as in terms of change over time. This study lays the groundwork for future studies that will seek to understand the relationship between behavioural change and neuroanatomical change in the brains of individuals who have experienced clinically transient cerebrovascular injuries.

## Methods

### Participants and clinical assessment

We recruited people who had TIAs or migraines from the Kingston Health Sciences Centre (Kingston, ON, Canada) stroke prevention clinic. Inclusion criteria that were common for both clinical groups were (1) the ability to understand the robotic task instructions, and (2) a lack of any upper-limb or other neurological injury that could impact performance on the robotic tests. Inclusion criteria specific for the TIA group were (1) a diagnosis of TIA, defined according to modern criteria^1^, and (2) a score of either (2a) zero or (2b) non-zero, but not pertaining to the relevant clinical event, on the National Institutes of Health Stroke Scale (NIHSS). The only specific inclusion criterion for the migraine group was a diagnosis of migraine following ICHD-3 criteria.

In addition to their initial assessments, individuals in the TIA group were invited to return at 6 weeks, 3 months, and 1 year after symptom resolution. People in the migraine group were invited back after 1 year only. All participants in this study provided written informed consent prior to taking part. This study was reviewed and approved by the Queen’s University Research Ethics Board, and was performed in accordance with the guidelines laid out by the Declaration of Helsinki.

### Robotic assessment

**Figure 5:**
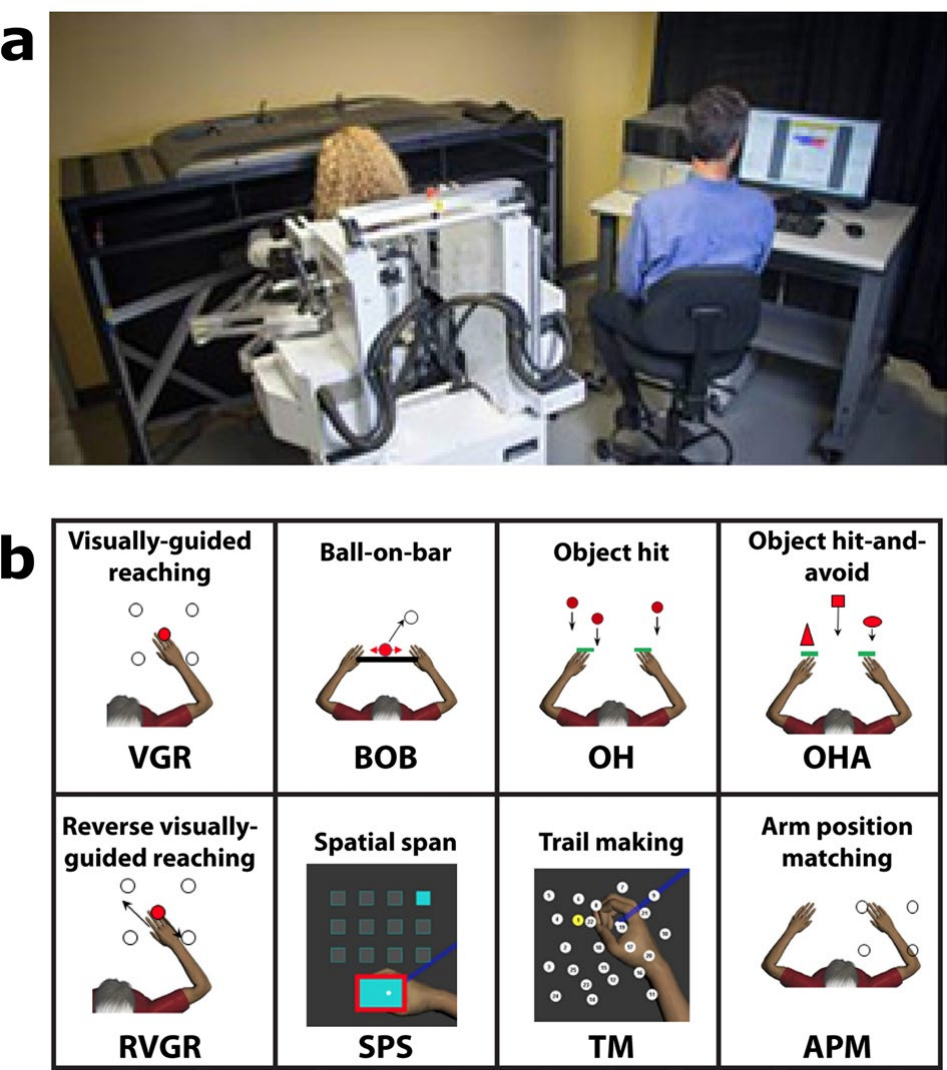
Kinarm and Kinarm Standard Tests (KSTs). (**a**) Kinarm Exoskeleton lab. (**b**) KSTs. Four tasks (VGR, BOB, OH, OHA) tested primarily motor skill, three tasks tested cognitive ability (RVGR, SPS, TM), and one task (APM) tested proprioception (a component of sensory ability).

Individuals in both the TIA and migraine groups completed a series of 8 behavioural tasks that tested motor, cognitive, and sensory domains using the Kinarm Exoskeleton Lab (Kinarm, Kingston, ON, Canada; see Fig. 5). This collection of behavioural tasks is referred to as the Kinarm Standard Tests™ (KSTs). See Supplemental Table S1 for detailed descriptions of the robotic tasks. Briefly, the Kinarm Exoskeleton required participants to be seated and to place their arms in troughs attached to the robotic linkage that also supported their weight against gravity. Participants moved their arms in the horizontal plane underneath a screen providing a virtual reality system aligned with this horizontal workspace. Visual feedback of the limbs was provided via the screen, usually with a white cursor dot (radius = 0.5 cm), sometimes with other shapes, or sometimes no feedback was provided.

There were 4 tasks to test primarily motor skills. Visually guided reaching (VGR) tested the participants’ ability to make quick and accurate reaches out and back to a series of either 8 or 4 peripheral targets^51^. The number of targets reduced during collection because the assessment time was substantially reduced, even though much of the same information is represented in the abbreviated version of the task^52^. Ball on bar (BOB) tested bimanual coordination by requiring participants to use both hands simultaneously to control a virtual ball balanced on a bar^53^. Object hit (OH) tested rapid motor planning by having individuals hit objects that were falling towards them on the screen, with the objective being to hit as many as possible before the end of the task^54^. Object hit and avoid (OHA) was almost the same as OH, except participants had to hit as many target objects away from them as possible while avoiding distractors^55^.

Three tasks placed additional emphasis on cognitive skills. Reverse visually guided reaching (RVGR) was similar to VGR, except that the hand had to be moved in the opposite direction to bring the cursor to the target, thus testing the ability to inhibit an automatic motor action^56^. Trail making (TM) was a robotic implementation of the classic neuropsychological assessment, the trail-making test, which assesses information processing speed and set-switching^57–59^. Spatial span (SPS) was similarly a robotic implementation of the Corsi block-tapping task, primarily testing spatial working memory^60^.

Finally, we tested sensory skill via proprioception. This was achieved using the arm position matching (APM) task, in which the robot moved one arm and the participant had to mirror-match the position using their other arm^61^.

### Statistical analysis

Performance in each robotic task was quantified using 10–20 spatial and temporal features of performance using a variety of disparate units (e.g., m/s, s, cm). These measures were transformed into standardized Z-scores (i.e., mean of 0, standard deviation of 1), referred to as “task parameters”, so that they could be compared and combined as necessary (See KST Summary, www.kinarm.com). Z-score transforms for each task parameter were generated based on performance of a large cohort of healthy controls and includes the influence of age, sex, handedness, and the type of robotic platform.

In addition to parameter Z-scores, we also calculated an aggregate metric referred to as “Task Score”, which is a single value that describes overall performance on a given task. Details of the process for going from multiple parameters’ Z-scores to a single Task Score are described in previous work^2, 28^ (see also KST Summary, www.kinarm.com). Briefly, Z-scores for each task parameter were all converted such that best performance was at zero and poorer performance was large positive. The root-sum square (RSS) of all task parameters was calculated for a large cohort healthy controls and this distribution was renormalized to a standard normal distribution (Z-Task Score). We then compressed the cumulative distribution function (CDF) of the Z-Task Scores to the right such that negative Z-Task Scores became close to zero but positive. Thus, the Task Score is an exclusively positive value with values close to 0 representing excellent performance and increasing values reflecting poorer overall performance on a task. The quantiles of the CDF of the Task Score distribution approximate those of the standard Normal distribution i.e., 68.3% of the area under the curve lies to the left of a Task Score of 1 and 95.4% lies to the left of a Task Score of 2.0. We define impairment as performance outside the envelope of 95% of healthy controls, corresponding to a Task Score of 1.96 or greater.

We previously quantified thresholds for significant change (SC) and expected learning effects (LE) for all task parameters and Z-Task Scores^28^. In order to calculate the boundaries for SC for the one-sided Task Scores, we performed a transform of the Z-Task Score to the Task Score as usual^2^, but with the addition or subtraction of the SC amount, creating a symmetric and curved SC boundary. LE were accounted for by calculating second assessment minus first assessment values, and identifying significant differences using a paired sample t-test. LE that were previously deemed significant were used in the present study. Our previous study identified that four tasks had significant LE: RVGR-D (− 0.72 expected change in Z-Task Score between first and second assessments), RVGR-ND (− 0.76), SPS (− 0.43), and TM (− 0.50). These were incorporated by subtracting the LE amount from Z-Task Score differences, and then performing the one-sided Task Score transform on the modified values, analogous to the approach with the SC values. Note that this makes the SC boundary asymmetrical. See Supplemental Fig. S1 for a graphical depiction of the consequences of this transformation.

We performed statistical analyses comparing Z-Task Scores in TIA and migraine groups at first and final assessments to the null hypothesis of a mean of 0, i.e., what is expected for the healthy control individuals (see previous sections on calculation of Z-scores for more details). We also tested the hypothesis of a *difference in means* of 0 between first and last assessments, to test for the significance of any observed changes over time. These comparisons were all performed by way of a 1-sample Student’s t-test on the differences between assessments. In order to correct for the 60 comparisons (10 total scores, 3 statistical tests, 2 timepoints), we set a significant p-value for these tests at ≈ 0.0077 using the false discovery rate (FDR) correction of Benjamini and Hochberg^62^.

We quantified the significance of changes between first- and last assessments at the individual level. Individual observation p-values were calculated by first fitting a Normal distribution using the mean and standard deviations (SDs) derived from healthy control Z-Task Score difference scores (final score minus initial score) that we reported on previously^47^, and then deriving the cumulative density function (CDF) of these distributions. The area under the curve was derived for each TIA or migraine Z-Task Score observation. For values with p > 0.5, we reported 1-CDF so that the reported probabilities were small values and not large (this has the same interpretation but is easier to plot). For example, if the control data difference scores for a given task had a mean of 0 and an SD of 1, then an observation at − 1.96 would have a p-value of ~ 0.025, whereas an observation at + 1.96 would have a p-value of ~ 0.975, which after subtracting from 1 would correspond to a p-value of ~ 0.025 as well.

We additionally used this individualized significant change approach to identify the p-values of changes between all assessments in the TIA cohort, in which 4 total assessments were performed. We simulated 1000 individual p-values for each transition between 1st and 2nd assessment, 2nd to 3rd, and 3rd to 4th, respectively, for all participants across all tasks. We used the FDR correction and determined that a p-value of approximately 1.3 × 10^–5^ was the threshold for significance. All statistical analyses were performed using SciPy v1.4.0 implemented in Python 3.7.1. Analysis code is publicly available on Github (https://github.com/8lers/TIA-kinarm-longitudinal).

### Reuse of data

Some of the TIA participant data in the present manuscript are reused from a previous paper^2^. We confirm that all 4 authors of the previous paper have provided written and informed consent for the data from the previous study to be used in the present paper.

## Supplementary Information

Supplementary Information.

## Data Availability

The datasets generated during and/or analyzed during the current study are available from the corresponding author on reasonable request.
