# Author Correction: Quantifying changes over 1 year in motor and cognitive skill after transient ischemic attack (TIA) using robotics

**DOI:** 10.1038/s41598-023-30417-1

**Published:** 2023-03-02

**Authors:** Leif E. R. Simmatis, Stephen H. Scott, Albert Y. Jin

**Affiliations:** 1 Toronto Rehabilitation Institute, University Health Network, Toronto, ON Canada; 2 Centre for Neuroscience Studies, Queen’s University, Kingston, ON Canada; 3 Department of Biomedical and Molecular Sciences, Queen’s University, Kingston, ON Canada; 4 Department of Medicine (Neurology), Queen’s University, Kingston, ON Canada

Correction to: *Scientific Reports* https://doi.org/10.1038/s41598-021-96177-y, published online 23 August 2021

The original version of this Article contained errors, where the authors found several values used for calculating the significant change thresholds and learning effects to be incorrect.

As a result, in the Results section, under the subheading ‘Individual longitudinal changes’,

“Furthermore, 3 individuals deteriorated and 2 improved in TM.”

now reads:

“Furthermore, 1 individual deteriorated and 0 improved in TM.”

And,

“Very few individuals in this group showed any significant change; 1 in VGR-ND, 2 in RVGR-ND, and 2 in TM.”

now reads:

“Very few individuals in this group showed any significant change; 1 in VGR-ND, 2 in RVGR-ND, and 0 in TM.”

Under the subheading ‘Patterns of significant change across tasks in TIA and Migraine’,

“Of these, 27 significant changes were observed, a rate of approximately 1/10 assessments. Furthermore, 16/28 (57%) individuals changed significantly on at least 1 behavioural task, and 8/28 (29%) changed significantly on at least 2 behavioural tasks. Of these, 7/28 (25%) significantly deteriorated on at least 1 task, whereas 13/28 (46%) significantly improved on 1 task. These changes were heterogeneous. For example, one individual in the TIA cohort significantly improved on both BOB and VGR-UA. Another significantly improved in VGR-UA and RVGR-UA, but significantly deteriorated on TM. In the migraine group, 23/210 total assessments demonstrated significant change between first and last test (approximately 1/10 individuals). Furthermore, 12/21 (57%) changed significantly on at least 1 behavioural task, and 8/21 (38%) changed significantly on at least 2 behavioural tasks. Of these, 6/21 (29%) significantly deteriorated on at least 1 behavioural task, and 9/21 (43%) significantly improved on at least 1 task. As in the TIA cohort, these changes were heterogeneous. For example, two individuals got better on RVGR-D, but deteriorated on OHA. Another significantly deteriorated on BOB and VGR-D.”

now reads:

“Of these, 23 significant changes were observed, a rate of approximately 1/12 assessments. Furthermore, 16/28 (57%) individuals changed significantly on at least 1 behavioural task, and 6/28 (21%) changed significantly on at least 2 behavioural tasks. Of these, 6/28 (21%) significantly deteriorated on at least 1 task, whereas 13/28 (46%) significantly improved on 1 task. These changes were heterogeneous. For example, one individual in the TIA cohort significantly improved on both BOB and RVGR-A. Another significantly improved in VGR-UA and RVGR-UA, but significantly deteriorated on TM. In the migraine group, 21/210 total assessments demonstrated significant change between first and last test (approximately 1/10 individuals). Furthermore, 12/21 (57%) changed significantly on at least 1 behavioural task, and 7/21 (33%) changed significantly on at least 2 behavioural tasks. Of these, 8/21 (38%) significantly deteriorated on at least 1 behavioural task, and 7/21 (33%) significantly improved on at least 1 task. As in the TIA cohort, these changes were heterogeneous. For example, one individual got better on RVGR-D, but deteriorated on OHA. Another significantly deteriorated on BOB and VGR-ND.”

Additionally, under the subheading ‘Individual change over multiple assessments in TIA’, the following two sentences were removed:

“For example, one individual in TM changed only approximately 0.6 Task Score units between 6 weeks and 3 months. However, because the starting score was very low (close to 0), this change was considered significant after FDR.”

Furthermore, in the Methods section, under the subheading ‘Statistical analysis’,

“Our previous study identified that four tasks had significant LE: RVGR-D (− 0.78 expected change in Z-Task Score between first and second assessments), RVGR-ND (− 0.67), SPS (− 0.39), and TM (− 0.23).”

now reads:

“Our previous study identified that four tasks had significant LE: RVGR-D (− 0.72 expected change in Z-Task Score between first and second assessments), RVGR-ND (− 0.76), SPS (− 0.43), and TM (− 0.50).”

Consequently, these errors have impacted Figures 2, 3 and 4. In Figure 2, the *p*-values and the curvature of several plotted lines were incorrect. In Figure 3, several of the significant improvements or deteriorations, indicated by blue or red cells respectively, were incorrect. Finally, in Figure 4, several of the significant differences between the assessments became non-significant as a result of the changes to the statistical boundaries used.

The original Figures 2, 3 and 4 and their respective accompanying legend appear below.

The original Article has been corrected.

**Figure 2:**
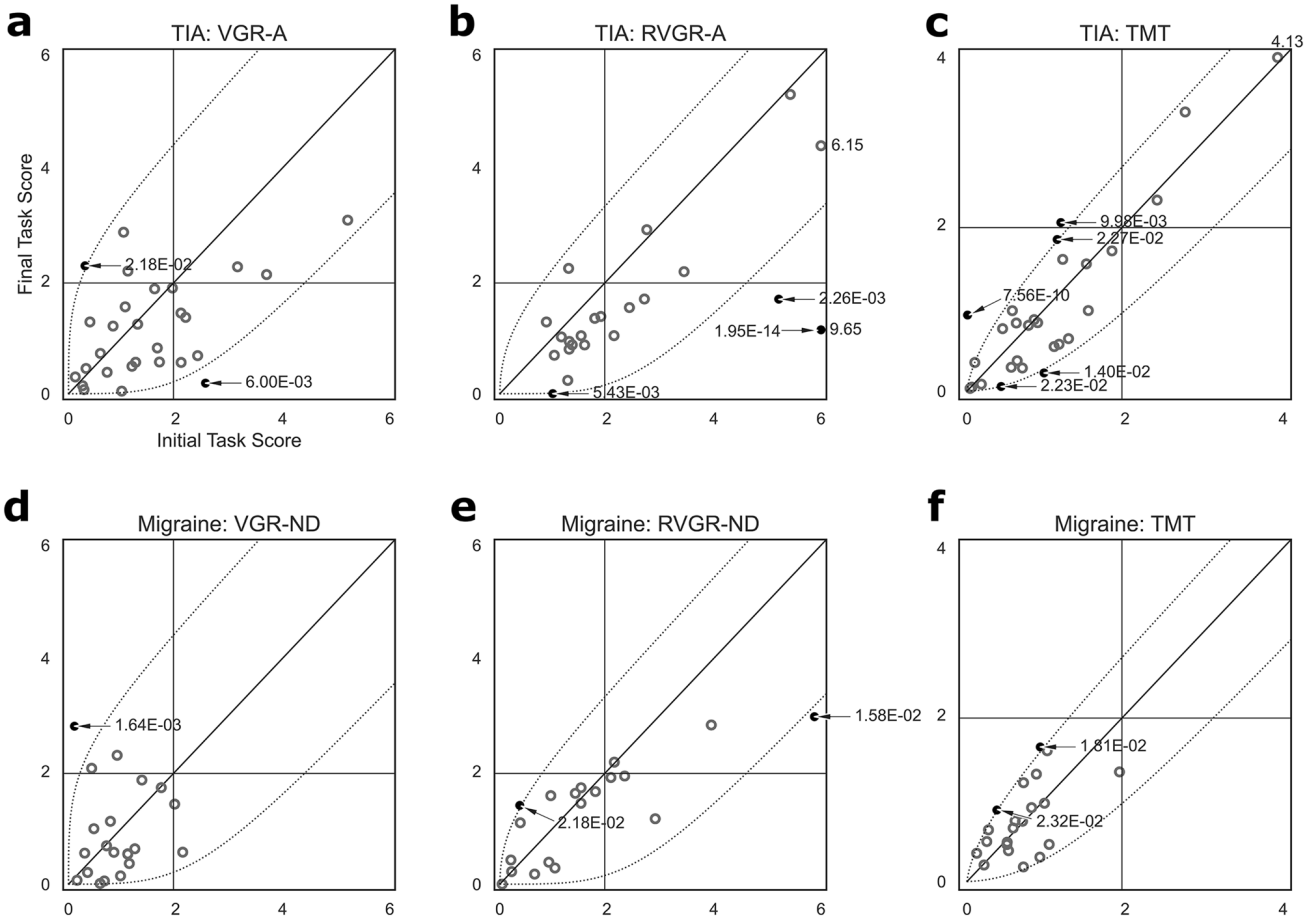
Scatterplots of one-sided Task Scores at first- and second assessments in TIA (**a**–**c**) and migraine (**d**–**f**) cohorts. All: Horizontal and vertical dashed lines indicate impairment thresholds (1.96); diagonal lines indicate unity. Curved dashed lines indicate thresholds of significant change based on the expected change of healthy control participants, accounting for learning effects in RVGR-A/RVGR-ND and TM only. Values > 6 (VGR-A/VGR-ND, RVGR-A/RVGR-ND) or > 4 (TM) are marked on the plots (no arrows pointing at relevant datapoints). Individual significant change p-values are marked when changes were significant (arrows pointing at relevant datapoints). Open darker circles represent non-significant changes (i.e., within the curved boundaries), whereas filled circles represent significant changes (i.e., outside the curved boundaries). (**a**–**c**) Plots for TIA. (**d**–**f**) Plots for migraine.

**Figure 3:**
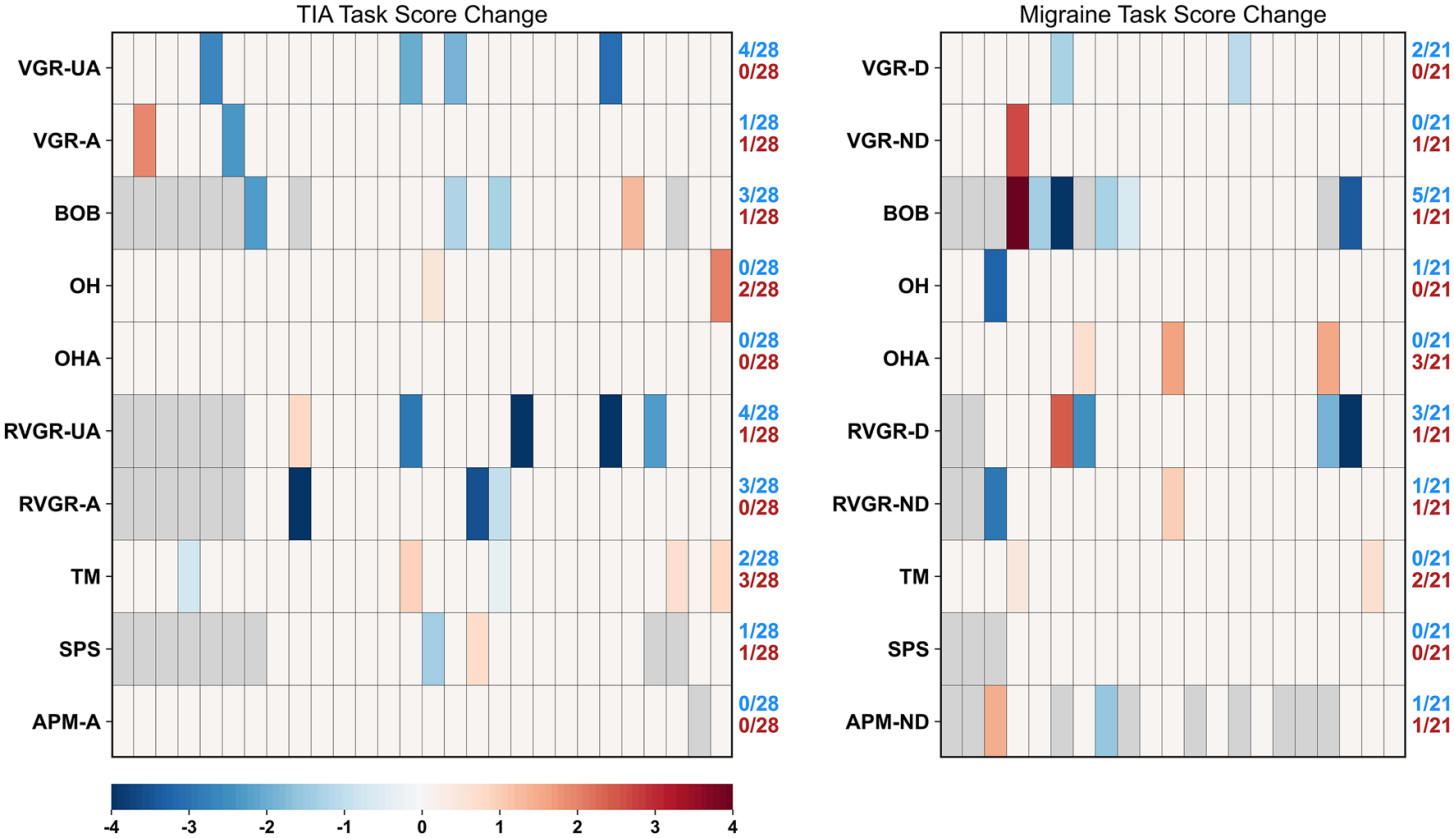
Heatmaps of significant changes in TIA and migraine groups between 2-weeks and 1-year. Participants are arrayed along the x-axes in no particular order. -*A* affected arm, -*UA* unaffected arm, -*D* dominant arm, -*ND* non-dominant arm. Blue cells represent significant lowering (improvement) in Task Score between 2 weeks and 1 year. Red cells represent significant increase (deterioration) in Task Score between 2 weeks and 1 year. Gray cells indicate missing values for those participants. Fractions improved and deteriorated are represented on the right-hand side of each axis for each task.

**Figure 4:**
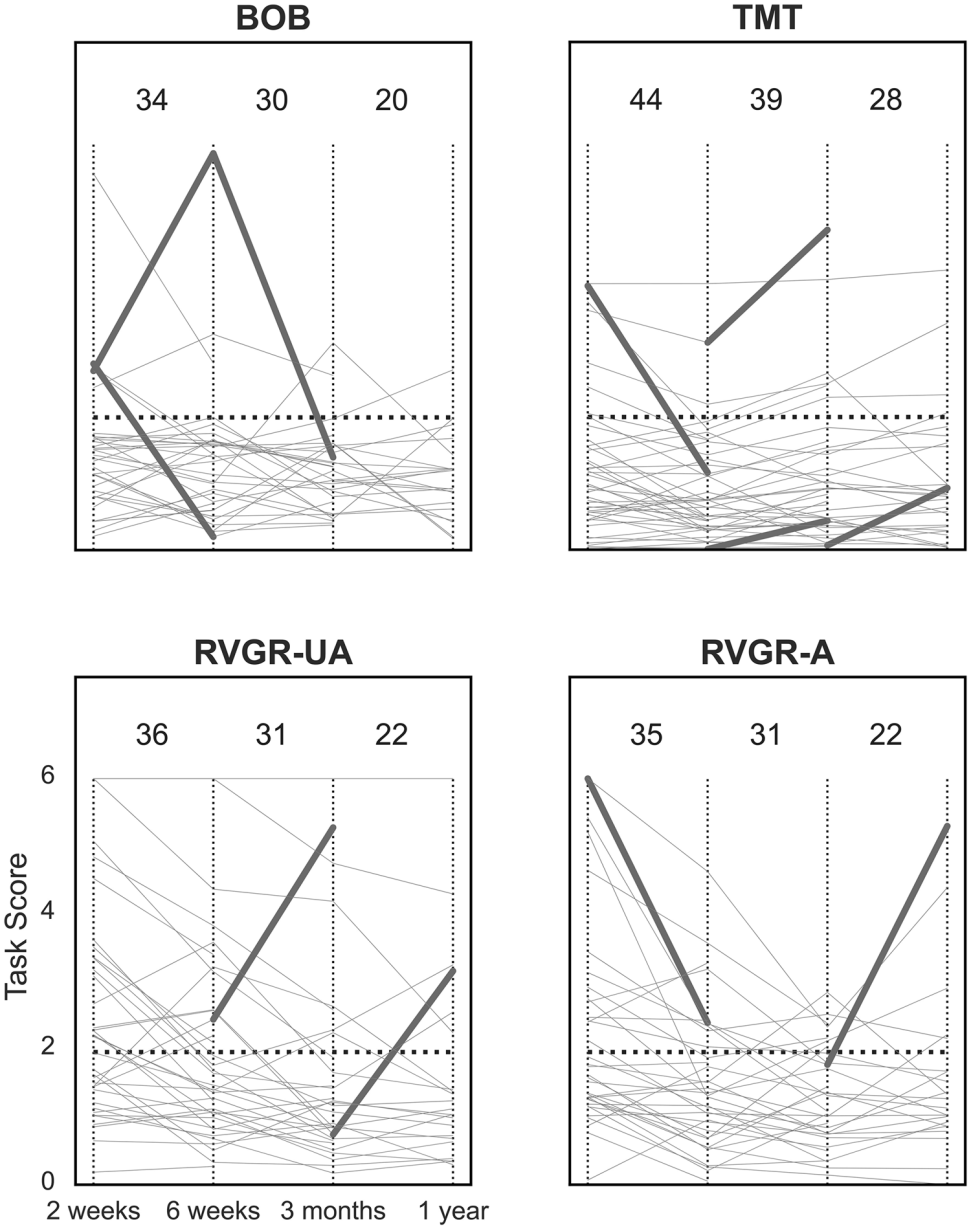
Line plots of task scores over time in the TIA cohort at all 4 assessments with significant changes corrected for the false discovery rate (FDR). *A* affected arm, *UA* unaffected arm. Thin vertical lines demarcate different assessment points for ease of viewing. Horizontal dashed lines represent the threshold for impairment in Task Scores (i.e., > 1.96). Thin grey lines represent each individual’s performance over time. Thick lines indicate significant improvement or deterioration between assessments. Values at the top of each plot indicate the total number of individuals plotted on the relevant interval, e.g., BOB between the first two vertical dashed lines has 34 individuals plotted, one of whom significantly improved, and one of whom significantly deteriorated.

