# Supplementary material for: Quantifying changes over 1 year in motor and cognitive skill after transient ischemic attack (TIA) using robotics
Source: Sci Rep. 2021 Aug 23;11:17011. doi: 10.1038/s41598-021-96177-y (PMC8382836; doi:10.1038/s41598-021-96177-y)
Supplement: Supplementary file 1 — Supplementary Information. [file 41598_2021_96177_MOESM1_ESM.docx]

**Supplemental Information**

Table S1: Task descriptions.

| **Task** | **Description** |
| --- | --- |
| **Visually guided reaching (VGR)** | The VGR task requires participants to reach quickly and accurately from a central target to each of 4 peripheral targets in sequence. This task tests the ability to make smooth and accurate reaches. During data collection the configuration changed from 8-targets to 4 for speed, however this had no effect on the data gathered [51,52]. |
| **Object hit (OH)** | The goal of the OH task is for participants to hit as many virtual balls away from them as possible. The task got harder as it went on, with balls falling more quickly [54]. This task tests bimanual motor skill. |
| **Object hit and avoid (OHA)** | In OHA, participants were instructed to hit two shapes (e.g. a vertical ellipse and a small square) and avoid the 6 other types, testing rapid decision-making processes [55]. This task is otherwise the similar to OH. |
| **Ball on bar (BOB)** | The BOB task requires participants to move a ball balanced on a bar to a each of 4 targets in sequence as quickly as possible. In level 1 the ball is fixed to the bar but in subsequent levels the ball is able to move but not fall off (level 2) or fall off easily (level 3). This task tests bimanual coordination [53]. |
| **Arm position matching (APM)** | APM test position sense. The robot moves one arm, and the participant is required to mirror-match the position using the other arm. This task requires accuracy, but speed is not tested [61]. |
| **Reverse visually guided reaching (RVGR)** | RVGR tests the ability to override automatic motor processes. A white cursor indicating the participant’s hand moves in the opposite direction of their hand [56], similar in presentation to VGR but with the exception of the added cognitive load. |
| **Trail making (TM)** | In TM version A, participants navigate to labelled targets with numbers (1..2..3.. and so on) as quickly as possible. In variant B, the targets are labelled with alternating numbers and letters (1..A..2..B..and so on), and the goal is the same. This task tests processing speed (A) and set-switching (B) [57–59]. |
| **Spatial span (SPS)** | A series of square targets is presented on a 3x4 grid and participants must recall them in the same order as shown to them, indicated by moving their cursor. After a successful trial, the sequence gets 1 target longer; after an unsuccessful sequence, it gets 1 target shorter. This task tests working memory capacity [60]. |


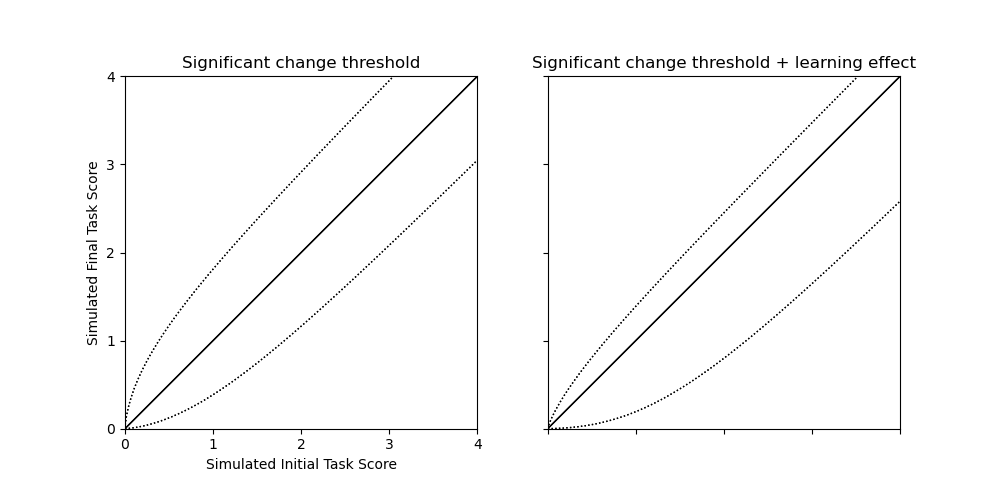


Figure S1. Plots of one-sided Task Scores depicting the effect of learning effects on the shape of significant change boundaries. Both plots: curved boundaries indicate significant change thresholds, and the central diagonal line is unity. Left plot: significant change threshold of ±1.0 on simulated Z-Task Scores. Right plot: the same significant change threshold of ±1.0, but with a learning effect of -0.5 incorporated.
